# Systematic pan-cancer analysis identifies cuproptosis-related gene DLAT as an immunological and prognostic biomarker

**DOI:** 10.18632/aging.204728

**Published:** 2023-05-17

**Authors:** Lidong Xu, Peipei Wu, Aimei Rong, Kunkun Li, Xingguo Xiao, Yong Zhang, Huili Wu

**Affiliations:** 1 Department of Gastroenterology, Zhengzhou Central Hospital Affiliated to Zhengzhou University, Zhengzhou 450000, China; 2 Medical Key Laboratory for Diagnosis and Treatment of Colorectal Cancer in Henan Province, Zhengzhou 450000, China; 3 Zhengzhou Key Laboratory for Diagnosis, Treatment and Research of Colorectal Cancer, Zhengzhou 450000, China; 4 Children’s Hospital Affiliated to Zhengzhou University, Zhengzhou 450000, China

**Keywords:** DLAT, pan-cancer, prognosis, immune infiltration, tumor microenvironment (TME)

## Abstract

Lipoylated dihydrolipoamide S-acetyltransferase (DLAT), the component E2 of the multi-enzyme pyruvate dehydrogenase complex, is one of the key molecules of cuproptosis. However, the prognostic value and immunological role of DLAT in pan-cancer are still unclear. Using a series of bioinformatics approaches, we studied combined data from different databases, including the Cancer Genome Atlas, Genotype Tissue-Expression, the Cancer Cell Line Encyclopedia, Human Protein Atlas, and cBioPortal to investigate the role of DLAT expression in prognosis and tumor immunity response. We also reveal the potential correlations between DLAT expression and gene alterations, DNA methylation, copy number variation (CNV), tumor mutational burden (TMB), microsatellite instability (MSI), tumor microenvironment (TME), immune infiltration levels, and various immune-related genes across different cancers. The results show that DLAT displays abnormal expression within most malignant tumors. Through gene set enrichment analysis (GSEA), we found that DLAT was significantly associated with immune-related pathways. Further, the expression of DLAT was also confirmed to be correlated with the tumor microenvironment and diverse infiltration of immune cells, especially tumor-associated macrophages (TAMs). In addition, we found that DLAT is co-expressed with genes encoding major histocompatibility complex (MHC), immunostimulators, immune inhibitors, chemokines, and chemokine receptors. Meanwhile, we demonstrate that DLAT expression is correlated with TMB in 10 cancers and MSI in 11 cancers. Our study reveals that DLAT plays an essential role in tumorigenesis and cancer immunity, which may be used to function as a prognostic biomarker and potential target for cancer immunotherapy.

## INTRODUCTION

Cancer is one of the most severe threats to public health, causing endless suffering for patients and their families, as well as imposing a heavy economic burden on society. To effectively prevent and control cancer, there needs to be a particular focus on elucidating the molecular mechanisms behind tumorigenesis, and exploring biological markers for cancer diagnosis and predicting treatment outcomes. The continuous creation and improvement of publicly accessible datasets, such as The Cancer Genome Atlas (TCGA), have made it possible to assess genes of interest in pan-cancer analyses using bioinformatics approaches.

The tumor microenvironment (TME) is a milieu that fosters tumorigenesis and comprises a diverse array of cell types and extracellular matrix [1]. It is well-established that immune cells are the key cellular constituents of TME, playing a pivotal role in the initiation and progression of human cancers. For instance, tumor-associated macrophages (TAMs), a critical immune cell population in TME, have been demonstrated to promote tumor progression by eliciting an immunosuppressive effect [2–5]. Immunotherapy has emerged as the fourth modality in cancer treatment following surgery, radiotherapy, and chemotherapy [6–10], with remarkable efficacy in treating various cancers. Among the most extensively studied immunotherapeutic agents are immune checkpoint inhibitors (ICIs), which have exhibited remarkable success in clinical management of malignant tumors. However, ICIs have limited efficacy in a fraction of cancer patients, with only 12.6% of all cancer patients deriving clinical benefit from their use [11]. Hence, the identification of biological markers that can elucidate the immunophenotype of TME and predict immune-related therapeutic targets is imperative.

Cuproptosis is a novel type of cell death that is caused by intracellular copper buildup and is characterized by the aggregation of mitochondrial lipoylated proteins and the instability of Fe-S cluster proteins, according to a recent study published in Science [12]. Modulating cuproptosis has the potential to be an effective therapeutic strategy for cuproptosis-sensitive tumors. Lipoylated dihydrolipoamide S-acetyltransferase (DLAT), a component E2 of the pyruvate dehydrogenase complex, is among the key molecules responsible for cuproptosis [12]. An earlier study indicated that the reduction of DLAT substantially diminished the proliferative ability of gastric cancer cells, revealing the therapeutic potential of DLAT in treating cancer [13]. Nevertheless, the expression profile and prognostic significance of DLAT, particularly the correlation between the expression patterns of DLAT and TME in pan-cancer, are still largely unexplored.

Here, based on various databases, we performed a comprehensive investigation on the DLAT expression and its prognostic landscape in pan-cancer. We also delved into the potential associations between DLAT expression and DNA promoter methylation, copy number variation (CNV), TME, immune infiltration levels, tumor mutational burden (TMB), and microsatellite instability (MSI). Furthermore, we carried out an enrichment analysis to gain further insight into the potential biological roles of DLAT in the development and progression of cancer. Our study’s findings strongly suggest that DLAT may play a critical role in the tumorigenesis and progression of various cancer types. As such, DLAT may have the potential to serve as a predictive and immunotherapeutic biological marker.

## RESULTS

### Pan-cancer analysis of DLAT expression

**Figure 1:**
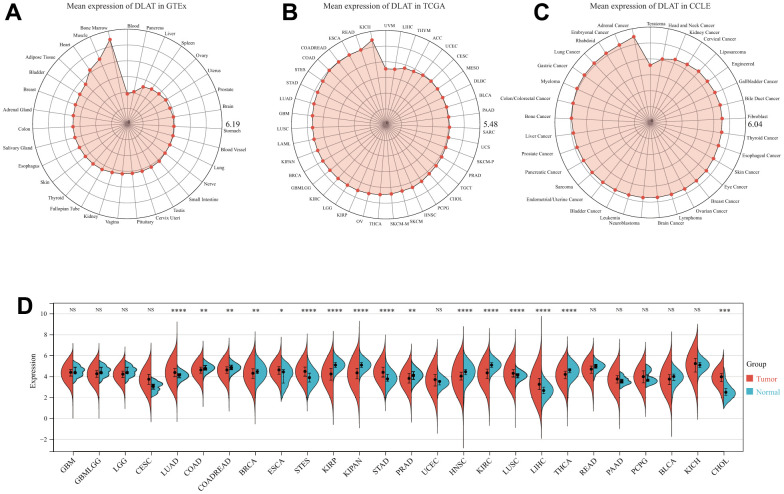
**DLAT mRNA expression levels in pan-cancer.** (**A**) DLAT expression levels in normal tissues from GTEx database. (**B**) DLAT expression levels in tumor tissues from TCGA database. (**C**) DLAT expression levels in tumor cell lines from CCLE database. (**D**) DLAT expression difference between tumor tissues and normal tissues from TCGA database; NS, no significance; *p < 0.05, **p < 0.01, ***p < 0.001, and ****p < 0.0001.

First, we utilized the Genotype Tissue-Expression (GTEx) dataset to evaluate the mRNA expression of DLAT in normal samples. Notably, the expression of DLAT was observed to be lowest in blood, as demonstrated in Figure 1A, while it was found to be highest in bone marrow. Furthermore, Figure 1B showed that the DLAT expression level was the lowest in uveal melanoma (UVM), while it was highest in kidney chromophobe (KICH) among the tumor tissues within the TCGA dataset. Subsequently, using the Cancer Cell Line Encyclopedia (CCLE) database of tumor cell lines, we determined that adrenal cancer had the highest DLAT gene expression (Figure 1C).

We also compared the tumor’s DLAT expression with that of normal tissues premised on TCGA data. Figure 1D demonstrates that DLAT expression was upmodulated in 7 distinct cancer types: cholangiocarcinoma (CHOL), esophageal carcinoma (ESCA), liver hepatocellular carcinoma (LIHC), lung adenocarcinoma (LUAD), lung squamous cell carcinoma (LUSC), stomach adenocarcinoma (STAD), and stomach and esophageal carcinoma (STES). Contrastingly, the expression of DLAT was shown to be low in nine different malignancies: breast invasive carcinoma (BRCA), colon adenocarcinoma (COAD), colon adenocarcinoma/rectum adenocarcinoma (COADREAD), head and neck squamous cell carcinoma (HNSC), pan-kidney cohort (KIPAN), kidney renal clear cell carcinoma (KIRC), kidney renal papillary cell carcinoma (KIRP), prostate adenocarcinoma (PRAD), and thyroid carcinoma (THCA).

Besides, we investigated whether a correlation existed between DLAT expression and tumor stages or grades. As per the findings, the expression of DLAT was significantly related to tumor stage in seven cancer types, namely, COADREAD, KIPAN, KIRC, LIHC, LUAD, rectum adenocarcinoma (READ), and THCA (Supplementary Figure 1). As for the DLAT expression in different tumor grades, significant differences were observed in five cancer types, including glioma (GBMLGG), KIPAN, KIRC, brain lower grade glioma (LGG), and pancreatic adenocarcinoma (PAAD) (Supplementary Figure 2).

**Figure 2:**
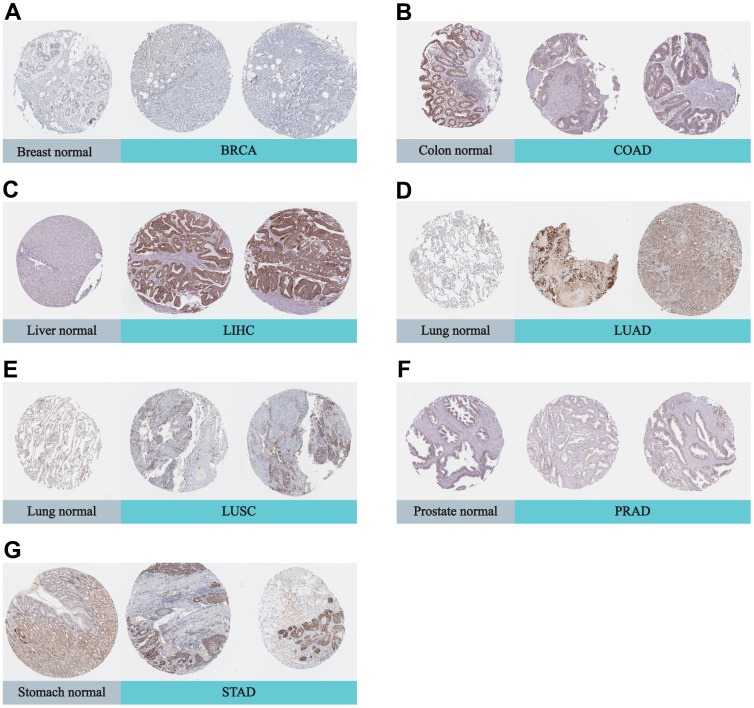
**Representative IHC in various normal (left) and tumor (right) tissues.** The protein expression of DLAT in (**A**) BRCA; (**B**) COAD; (**C**) LIHC; (**D**) LUAD; (**E**) LUSC; (**F**) PRAD; (**G**) STAD.

In addition, we utilized the Human Protein Atlas (HPA) database to compare the expression of DLAT in normal and tumor samples at the protein level. Strong staining of DLAT was observed in STAD tissues, in contrast to the normal stomach, which had only weak immunohistochemistry (IHC) staining (Figure 2). DLAT staining was found to be moderate in samples of normal liver tissue, but the staining was strong in samples of tumor tissues. The DLAT staining in normal lung tissues was weak, whereas that of LUSC tissues was moderate and strong in LUAD tissues. Conversely, DLAT staining was strong in samples of normal colon and prostate tissues but only moderate in tumor tissue samples. DLAT staining was moderate in normal breast tissue samples but weak in cancer tissues.

### Prognostic relevance of DLAT

**Figure 3:**
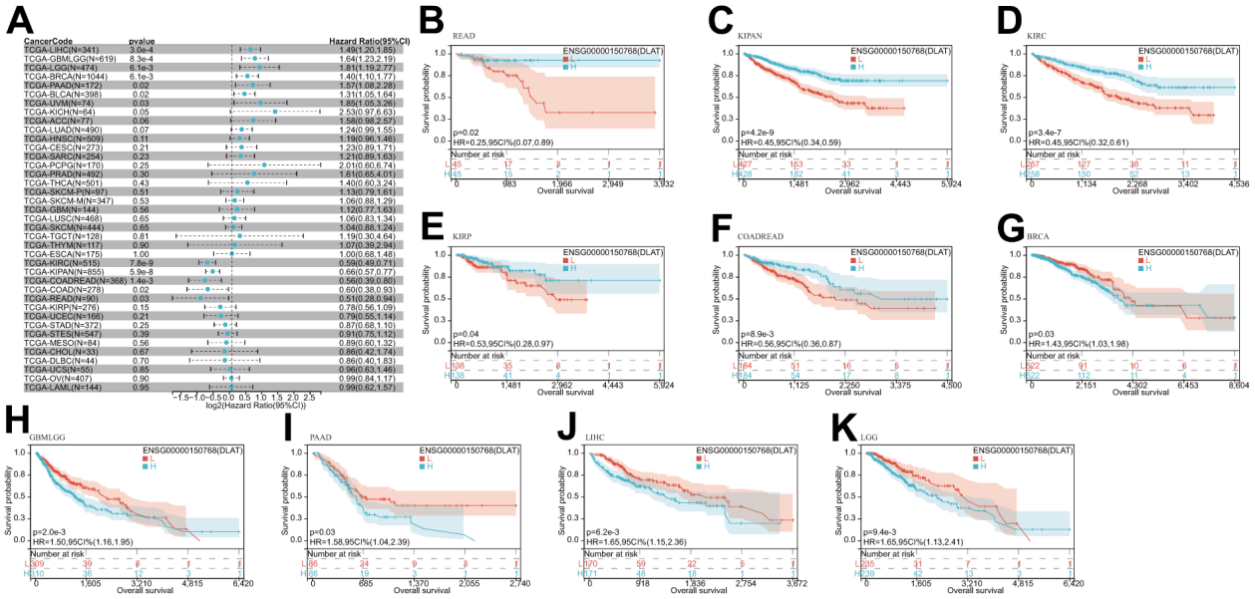
**Relationship of DLAT expression with patient OS.** (**A**) Forest map shows the univariate Cox regression analysis results for DLAT in TCGA pan-cancer samples. (**B**–**K**) Kaplan–Meier OS curves of DLAT expression in the ten significantly associated tumors.

**Figure 4:**
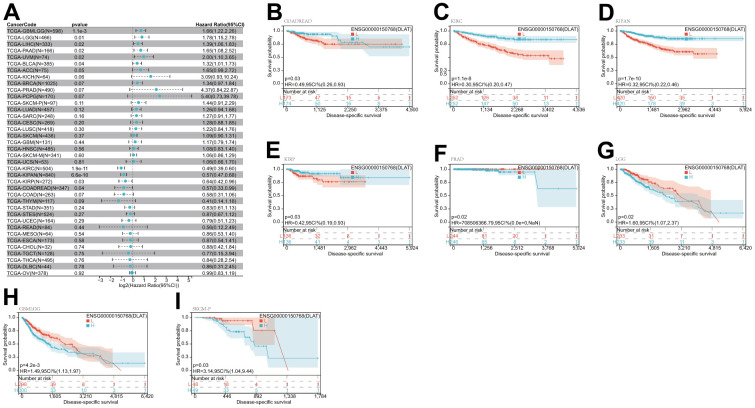
**Relationship of DLAT expression with patient DSS.** (**A**) Forest map shows the univariate Cox regression analysis results for DLAT in TCGA pan-cancer samples. (**B**–**I**) Kaplan–Meier DSS curves of DLAT expression in the eight significantly associated tumors.

**Figure 5:**
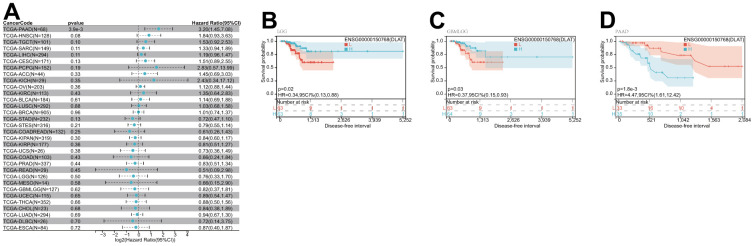
**Relationship of DLAT expression with patient DFI.** (**A**) Forest map shows the univariate Cox regression analysis results for DLAT in TCGA pan-cancer samples. (**B**–**D**) Kaplan–Meier DFI curves of DLAT expression in the three significantly associated tumors.

**Figure 6:**
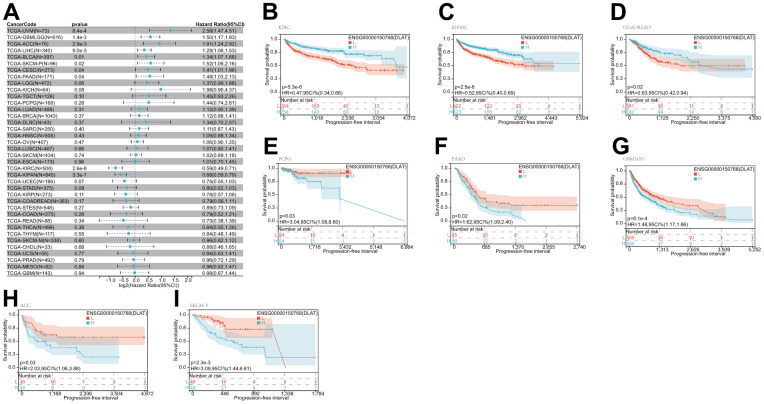
**Relationship of DLAT expression with patient PFI.** (**A**) Forest map shows the univariate Cox regression analysis results for DLAT in TCGA pan-cancer samples. (**B**–**I**) Kaplan–Meier PFI curves of DLAT expression in the eight significantly associated tumors.

After that, we examined the role of DLAT expression in tumor survival outcomes. For overall survival (OS), DLAT functioned as a risk factor in bladder urothelial carcinoma (BLCA), BRCA, GBMLGG, LGG, LIHC, PAAD, and UVM based on the analyses of Cox regression. In contrast, it functioned as a protective factor in COAD, COADREAD, KIPAN, KIRC, and READ. The Kaplan-Meier (KM) analysis on the OS illustrated that high levels of DLAT expression were related to dismal prognoses in patients suffering from BRCA, GBMLGG, LGG, LIHC, and PAAD, but a favorable prognosis was found in COADREAD, KIPAN, KIRC, KIRP, and READ (Figure 3B–3K). Subsequently, Cox regression analysis of the disease-specific survival (DSS) showed that DLAT was a risk factor for six distinct cancers, including BLCA, GBMLGG, LGG, LIHC, PAAD, and UVM. On the other hand, as shown in Figure 4A, it was a protective factor in COADREAD, KIPAN, KIRC, and KIRP. Figure 4B–4E show that a high level of DLAT expression was associated with a favorable DSS for the COADREAD, KIPAN, KIRC, and KIRP based on the results of KM analysis. However, poor DSS was found in patients with high DLAT expression levels in GBMLGG, LGG, PRAD, and primary skin cutaneous melanoma (SKCM-P) (Figure 4F–4I). Additionally, the findings result from Cox regression analysis showed that DLAT was a risk factor for disease-free interval (DFI) in PAAD (Figure 5A). Further KM analysis demonstrated that patients with PAAD whose DLAT expression was higher exhibited a poorer DFI in contrast with those whose DLAT expression was lower, and reversely in GBMLGG and LGG (Figure 5B–5D). Lastly, for progression-free interval (PFI), DLAT served as a risk factor in patients with adrenocortical carcinoma (ACC), BLCA, cervical squamous cell carcinoma and endocervical adenocarcinoma (CESC), GBMLGG, LIHC, PAAD, SKCM-P, and UVM but was found to be a protective factor in KIPAN and KIRC according to the Cox regression analysis findings (Figure 6A). Furthermore, an unfavorable PFI was found in patients exhibiting high levels of DLAT expression in ACC, GBMLGG, PAAD, pheochromocytoma and paraganglioma (PCPG), and SKCM-P based on the results of KM analysis, while opposite results were found in COADREAD, KIPAN, and KIRC, as depicted in Figure 6B–6I.

### Genetic alteration of DLAT

**Figure 7:**
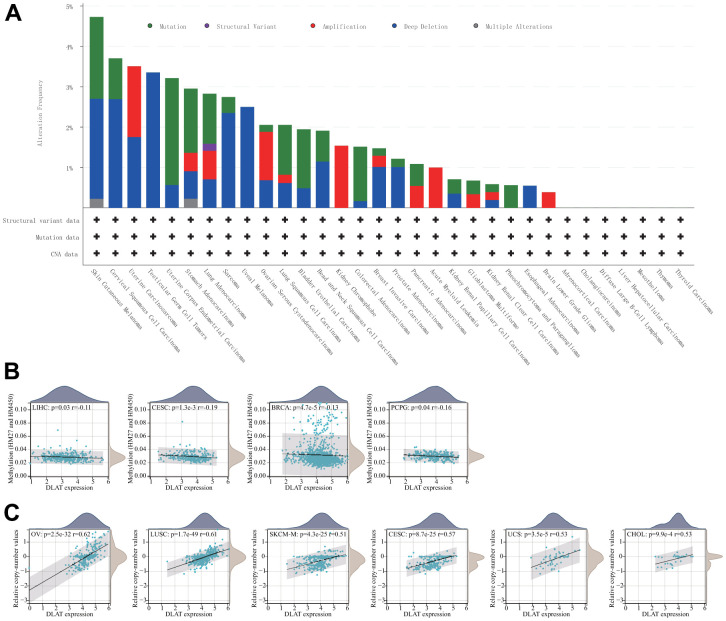
**Relationship of DLAT expression with gene alterations.** (**A**) The genetic alteration type and frequency of DLAT in various cancers. (**B**) Correlation between DLAT expression and gene promoter methylation in LIHC, CESC, BRCA, and PCPG. (**C**) Correlation between DLAT expression and copy number variation in OV, LUSC, SKCM-M, CESC, UCS, and CHOL.

Both DNA methylation and genetic alterations have been shown to be associated with tumorigenesis and tumor progression. First, we evaluated the frequency of DLAT alterations in cancer patients utilizing the cBioPortal database. Notably, patients with SKCM had the highest frequency of DLAT alterations, accounting for 4.73 percent, in comparison to other types of cancer. The “deep deletion” type was prevalent in most cancers, including BRCA, ESCA, HNSC, KIRC, KIRP, PRAD, sarcoma (SARC), skin cutaneous melanoma (SKCM), testicular germ cell tumors (TGCT), uterine carcinosarcoma (UCS), and UVM (Figure 7A). Subsequently, we assessed the degree of association between DLAT mRNA expression and promoter methylation and found significant correlations in seven malignancies (Table 1). Interestingly, an inverse relationship was observed between promoter methylation level and DLAT expression level in BRCA, CESC, LIHC, and PCPG (Figure 7B). Furthermore, the relationship between DLAT expression level and CNV was also analyzed. The results indicated that DLAT expression levels were positively linked to CNV in nearly all cancer types, except for GBMLGG, acute myeloid leukemia (LAML), LGG, and UVM (Table 1). Figure 7C displays the six tumor types with the highest correlation coefficients.

**Table 1:** Correlation of DLAT expression with methylation and copy number variation analysis.

**Cancer**	**Methylation**		**Copy number variation**	
	**Correlation**	**P-value**	**Correlation**	**P-value**
ACC	-0.110676692	0.340450099	0.241333091	0.038320817
BLCA	-0.056523669	0.257005235	0.302098079	6.60E-10
BRCA	-0.125524108	4.68E-05	0.44713125	1.07E-52
CESC	-0.191419956	0.001262602	0.565240567	8.72E-25
CHOL	0.06975547	0.685070584	0.525712824	0.000992151
COAD	0.121855797	0.04465066	0.362598837	6.61E-10
COADREAD	0.086500499	0.10274131	0.386362669	3.15E-14
DLBC	0.00141844	0.99283036	0.294088655	0.047275298
ESCA	-0.064033867	0.395785955	0.438311851	9.41E-10
GBM	0.037407919	0.681232625	0.318860724	9.27E-05
GBMLGG	0.003633969	0.927756669	0.026102661	0.5084603
HNSC	0.021295869	0.630696138	0.512534841	3.06E-35
KICH	0.196809441	0.115990398	0.295144747	0.016995871
KIPAN	0.010182856	0.767287846	0.197908613	6.55E-09
KIRC	-0.074889367	0.093065046	0.102378405	0.021782614
KIRP	0.118689702	0.048038258	0.178113659	0.002880287
LAML	0.0244985	0.77547768	-0.046214963	0.590402807
LGG	0.061100855	0.171672007	0.001793117	0.968129188
LIHC	-0.114178401	0.030782778	0.263528352	5.09E-07
LUAD	0.076078952	0.091860516	0.339220432	1.09E-14
LUSC	0.086387675	0.060470486	0.609239853	1.71E-49
MESO	-0.047186082	0.66804556	0.498525197	1.20E-06
OV	0.021515816	0.712389737	0.620199298	2.54E-32
PAAD	0.151578403	0.044621541	0.250753808	0.000816834
PCPG	-0.155741128	0.039014995	0.325035724	2.75E-05
PRAD	-0.009694302	0.830337362	0.199105752	9.76E-06
READ	0.026441403	0.810167295	0.458338446	9.09E-06
SARC	-0.092194104	0.144463404	0.37677172	7.47E-10
SKCM	0.017400464	0.721209537	0.514979865	4.25E-25
SKCM-M	-0.026379782	0.623332758	0.514979865	4.25E-25
SKCM-P	0.154626503	0.188365685	-	-
STAD	-0.045375971	0.373969159	0.366014768	1.29E-13
STES	-0.039599163	0.347879754	0.343190951	5.60E-17
TGCT	0.155453269	0.076230992	0.311966006	0.000286343
THCA	0.085506319	0.057293008	0.160056081	0.000359953
THYM	-0.110290619	0.236499307	0.313489255	0.000578116
UCEC	0.079839184	0.2935886	0.251401653	0.000790894
UCS	0.092141014	0.499416915	0.527462617	3.51E-05
UVM	0.062464626	0.584459231	-0.14763121	0.194154266

### Gene set enrichment analyses (GSEA) of DLAT

**Figure 8:**
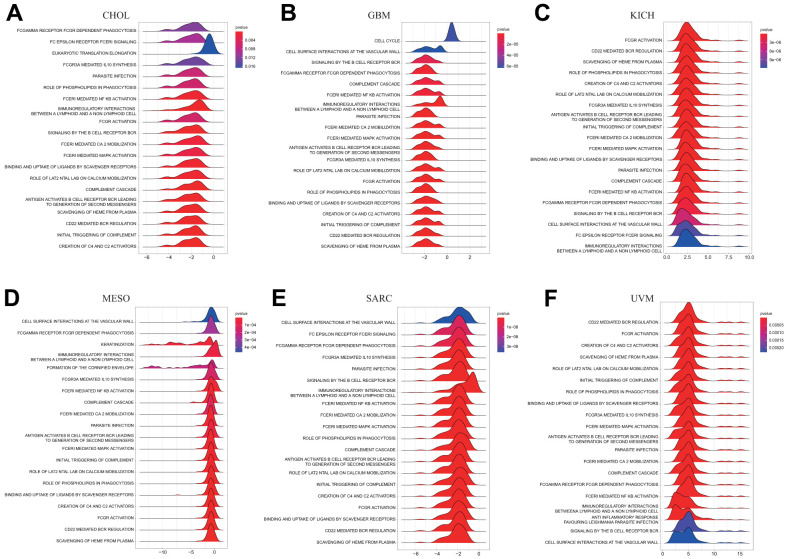
**GSEA of DLAT in pan-cancer.** (**A**–**F**) TOP20 GSEA terms in indicated tumor types.

We conducted a GSEA analysis to identify the pathways in which DLAT may be involved, in order to better understand the potential biological processes underlying DLAT expression in different cancer tissues. Interestingly, our results showed that several types of tumors exhibited similar GSEA results. The findings demonstrated that DLAT is implicated in immune modulation-associated pathways in pan-cancer, such as “Antigen activates B Cell Receptor (BCR) leading to generation of second messengers,” “CD22 mediated BCR regulation,” “FCGR activation,” “FCGR3A-mediated IL10 synthesis,” “FCERI mediated Ca+2 mobilization,” “FCERI mediated MAPK activation,” “complement cascade,” “Role of LAT2/NTAL/LAB on calcium mobilization,” and “Immunoregulatory interactions between a Lymphoid and a non-Lymphoid cell” (Figure 8A–8F). As per these findings, DLAT possibly performs an instrumental function in modulating cancer immunity.

### Tumor microenvironment analysis

**Figure 9:**
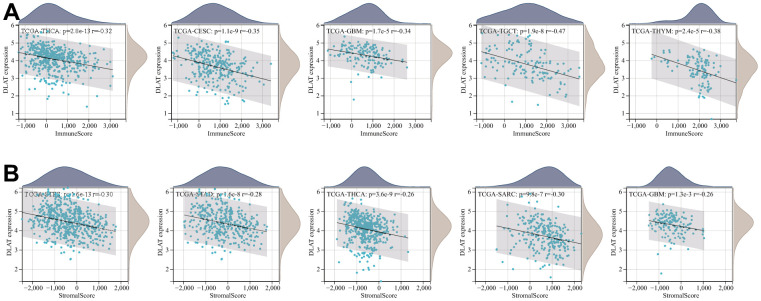
**Five tumors with the highest correlation coefficients between DLAT expression and the tumor microenvironment.** (**A**) Correlation between DLAT and immune scores in THCA, CESC, GBM, TGCT, and THYM. (**B**) Correlation between DLAT and stromal scores in STES, STAD, THCA, SARC, and GBM.

The TME, which enables the survival of tumor cells, plays a crucial role in the development and progression of cancers. Therefore, we utilized the immune and stromal scores derived from the ESTIMATE algorithm to assess any potential associations between DLAT expression and TME components. The result revealed a negative correlation between DLAT expression and the immune score across 27 different cancer types, including ACC, BRCA, CESC, ESCA, glioblastoma multiforme (GBM), GBMLGG, HNSC, LAML, LGG, LUAD, LUSC, mesothelioma (MESO), KIPAN, KIRC, KIRP, PCPG, PRAD, SARC, SKCM, metastatic skin cutaneous melanoma (SKCM-M), SKCM-P, STAD, STES, TGCT, THCA, thymoma (THYM), and uterine corpus endometrial carcinoma (UCEC). Moreover, in 14 different types of cancer, including ACC, CESC, ESCA, GBM, KIPAN, LUAD, LUSC, PCPG, SARC, SKCM-P, STAD, STES, THCA, and UCEC, DLAT expression was found to be inversely related to stromal scores (Table 2). The five tumor types with the highest correlation coefficients are shown in Figure 9.

**Table 2:** Correlation of DLAT expression with ImmuneScore and StromalScore analysis.

**Cancer**	**StromalScore**		**ImmuneScore**		**ESTIMATEScore**	
	**Correlation**	**P-value**	**Correlation**	**P-value**	**Correlation**	**P-value**
ACC	-0.22606341	0.048050107	-0.285135917	0.011952376	-0.252458068	0.026753897
BLCA	-0.063025771	0.205617812	-0.034801932	0.484913174	-0.055278109	0.26705898
PBRCA	-0.040361711	0.185642961	-0.087674174	0.003983468	-0.078662127	0.009808363
CESC	-0.211804412	0.000273771	-0.34759274	1.09E-09	-0.321947576	1.92E-08
CHOL	0.094465894	0.583678048	0.01981982	0.908656611	0.029086229	0.866273481
COAD	-0.022360485	0.708497089	0.000815502	0.989122154	-0.003079804	0.958935781
COADREAD	-0.006338452	0.902894109	0.007465967	0.885729628	0.008446876	0.870839978
DLBC	0.107246377	0.47806855	0.160283688	0.287293456	0.19617638	0.191329931
ESCA	-0.203472479	0.006009245	-0.219845759	0.002942335	-0.231813299	0.001689439
GBM	-0.258238242	0.001317428	-0.340844067	1.73E-05	-0.316438383	7.14E-05
GBMLGG	0.039216593	0.315906668	-0.104045309	0.007652721	-0.052348479	0.180526254
HNSC	0.049588065	0.260386273	-0.095396271	0.030099129	-0.037001231	0.401148652
KICH	0.01097028	0.930884797	-0.042701049	0.735558316	-0.030113636	0.811782533
KIPAN	-0.09741126	0.003862715	-0.259027828	6.30E-15	-0.193235856	7.83E-09
KIRC	-0.02281721	0.600887211	-0.228353489	1.13E-07	-0.159295367	0.000237713
KIRP	-0.087456752	0.140811846	-0.278170894	1.84E-06	-0.219835462	0.000183433
LAML	-0.06546568	0.42764272	-0.265934937	0.001045289	-0.192147057	0.018893787
LGG	-0.001456	0.973988806	-0.157260508	0.000394408	-0.107287611	0.015970944
LIHC	0.055516993	0.291468135	-0.098397159	0.061096174	-0.02983202	0.57102532
LUAD	-0.090894073	0.04219688	-0.259229816	4.03E-09	-0.19432317	1.21E-05
LUSC	-0.161180812	0.000335921	-0.239839405	7.46E-08	-0.21887755	9.72E-07
MESO	-0.184024741	0.091808359	-0.27431246	0.011068649	-0.275250514	0.010785806
OV	-0.03876112	0.429851836	-0.029096574	0.553508612	-0.036183587	0.461175168
PAAD	0.229962393	0.002075934	0.080041681	0.289581302	0.159242606	0.034251107
PCPG	-0.244389176	0.001044702	-0.200027376	0.007599406	-0.233595748	0.001752961
PRAD	-0.050393518	0.263113886	-0.103080446	0.021807161	-0.082292737	0.067343782
READ	0.037856497	0.721643219	0.024574075	0.817146987	0.032561047	0.759300123
SARC	-0.299247798	9.78E-07	-0.298479507	1.05E-06	-0.328150775	6.84E-08
SKCM	-0.091078395	0.052988477	-0.168367845	0.000323973	-0.146244898	0.001824685
SKCM-M	-0.101957095	0.056348902	-0.204065566	0.00011806	-0.177121633	0.000859379
SKCM-P	-0.203997717	0.040736372	-0.216344978	0.02978175	-0.22232641	0.025444485
STAD	-0.281993141	1.59E-08	-0.253876425	4.02E-07	-0.302226321	1.23E-09
STES	-0.302810393	1.57E-13	-0.292118776	1.17E-12	-0.324426144	2.06E-15
TGCT	-0.01361066	0.876904541	-0.465363518	1.89E-08	-0.365338082	1.65E-05
THCA	-0.259326531	3.57E-09	-0.317925606	2.82E-13	-0.313422311	6.30E-13
THYM	-0.031484647	0.735021117	-0.378243054	2.41E-05	-0.279253221	0.002196153
UCEC	-0.238027376	0.001377047	-0.305000769	3.48E-05	-0.301049392	4.44E-05
UCS	-0.011791042	0.93126854	-0.144636785	0.287530181	-0.100138417	0.462757069
UVM	0.075062227	0.510883902	0.132049636	0.246022963	0.125489146	0.270480543

### Analysis of immune cell infiltration

**Figure 10:**
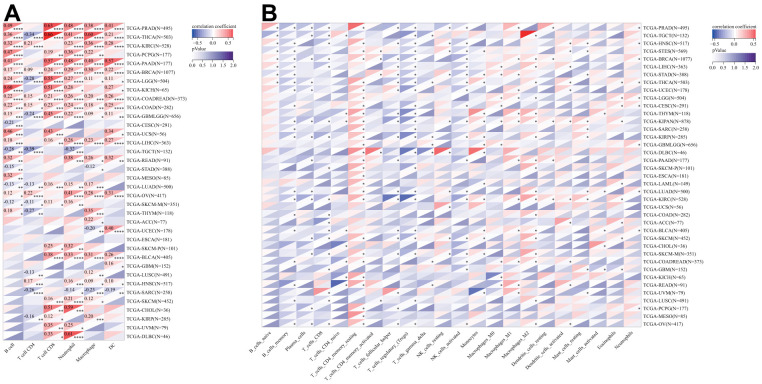
**Relationship of DLAT expression with Immune cell infiltration analysis.** (**A**) The relationship between DLAT expression levels and the levels of infiltration of 6 immune-related cells by using TIMER algorithm. (**B**) The relationship between DLAT expression levels and the levels of infiltration of 22 immune-related cells by using CIBERSOFT algorithm.

Following this, we conducted an inquiry to examine the potential relationship between DLAT expression and the degree of infiltration by various immune cells. According to the results from the TIMER algorithm, DLAT expression had a significant correlation with the levels of infiltrating neutrophils in 25 different cancer types, macrophages in 24, B cells in 22, CD8+T cells in 21, dendritic cells in 19, and CD4+T cells in 16 (Figure 10A). Furthermore, we found that the six cancer types with the strongest association between DLAT expression and immune infiltration levels were BRCA, COAD, COADREAD, GBMLGG, LGG, and THCA. In the case of BRCA, COAD, and COADREAD, all six immune cell types were positively linked with DLAT expression. As for GBMLGG, LGG, and THCA, B cells, CD8+T cells, macrophages, neutrophils, and dendritic cells were positively correlated with DLAT expression, whereas CD4+T cells showed a negative correlation.

Consistent with the aforementioned findings, further analyses employing the CIBERSORT algorithm corroborated that DLAT expression was significantly associated with immunological infiltration levels in most cancer types (Figure 10B). Furthermore, there were statistically significant links between the levels of DLAT expression and the various subtypes of infiltrating macrophages. DLAT expression exhibited a positive correlation with the levels of M0 macrophage infiltration in GBM, GBMLGG, LGG, LIHC, STAD, and STES, while showing a reverse correlation in KIPAN, KIRC, and UVM. DLAT expression was positively linked to the macrophage M1 subtype in BLCA, BRCA, COADREAD, KIPAN, KIRC, LGG, PAAD, PRAD, READ, STAD, and THYM, while showing a negative correlation in GBM and THCA. The degree of infiltrating macrophage M2 was negatively associated with DLAT expression only in LGG, whereas it was positively associated with 19 cancer types, including COAD, COADREAD, BRCA, HNSC, KIPAN, KIRC, KIRP, LIHC, LUAD, LUSC, ovarian serous cystadenocarcinoma (OV), PAAD, SKCM, SKCM-M, SKCM-P, STAD, TGCT, THCA, and THYM.

### Analyses of tumor mutational burden and microsatellite instability

**Figure 11:**
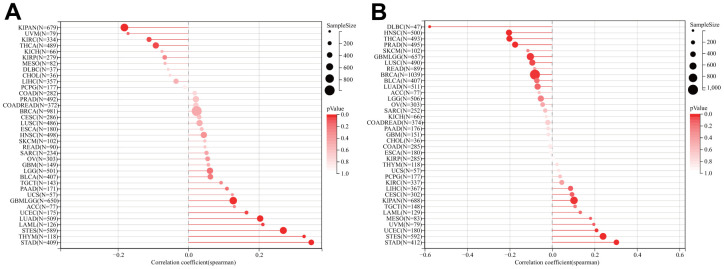
**Relationship of DLAT expression and TMB and MSI.** (**A**) Lollipop chart illustrating the relationship between DLAT expression and TMB. (**B**) Lollipop chart illustrating the relationship between DLAT expression and MSI.

TMB and MSI have proven to be effective predictors of immune treatment responses across a multitude of tumors. We subsequently investigated whether DLAT expression correlated with TMB and MSI in various cancers (Figure 11). The results indicated a significant correlation between DLAT expression and TMB in ten different types of cancer. Specifically, DLAT expression was positively correlated with TBM in GBMLGG, LAML, LUAD, STES, STAD, THYM, and UCEC, but inversely correlated with TMB in KIPAN, KIRC, and THCA (Figure 11A). Additionally, we found that DLAT expression was positively correlated with MSI in KIPAN, STAD, STES, and UCEC, but negatively correlated with MSI in BRCA, lymphoid neoplasm diffuse large B-cell lymphoma (DLBC), GBMLGG, HNSC, LUSC, PRAD, and THCA (Figure 11B).

### Immune-related genes analyses

**Figure 12:**
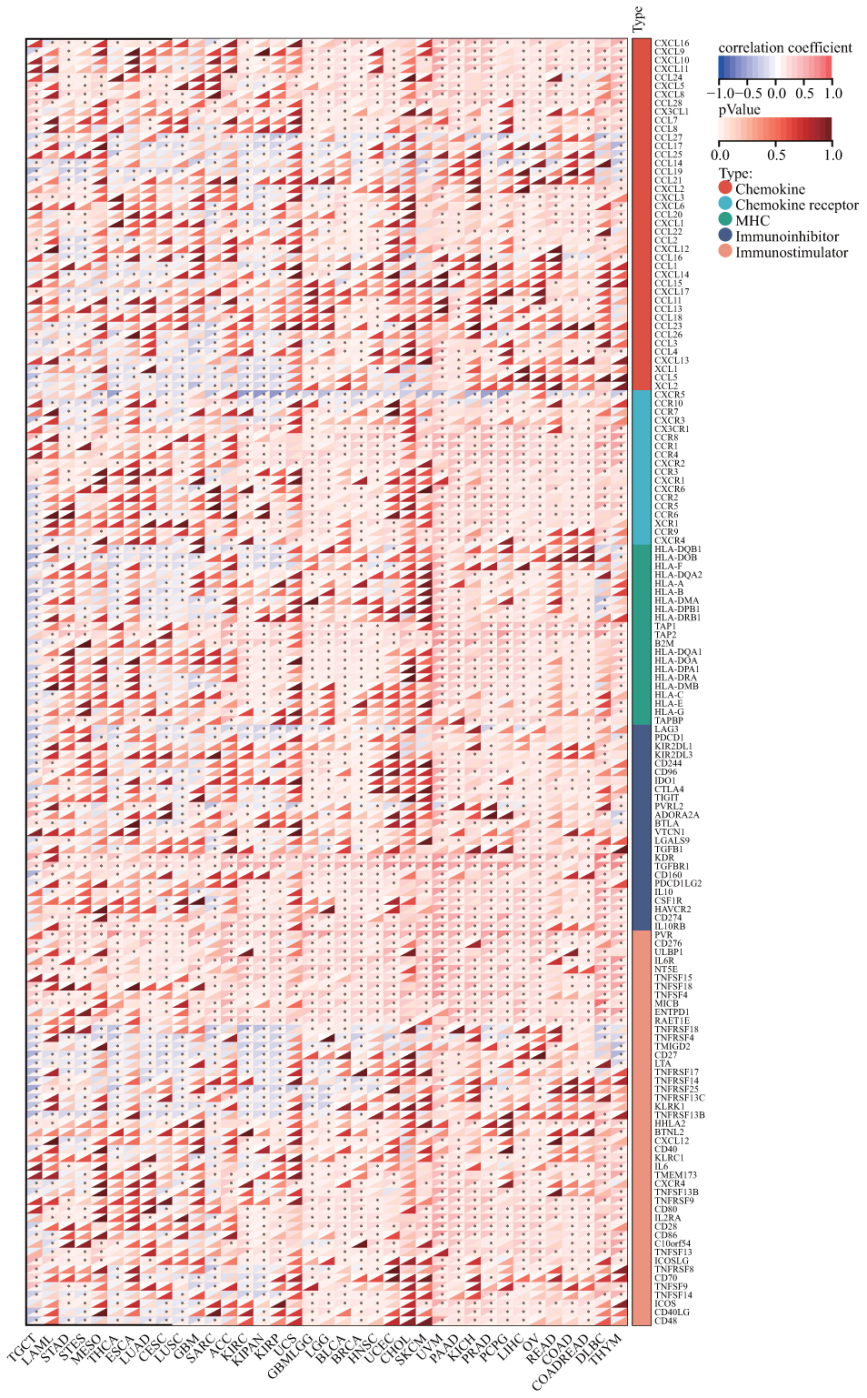
**Co-expression of DLAT with immune-associated genes.** *p < 0.05.

We further investigated whether there existed a co-expression between DLAT and immune-related genes in a diverse array of cancers. The results indicate that DLAT was closely associated with the majority of immune-related genes, as evidenced by Figure 12. Chemokines, such as CCL28, CXCL8, and CXCL16, and chemokine receptors, including CCR1, CCR8, and CXCR2, were positively linked to DLAT expression in various cancers. Notably, there was a robust relationship between the expression of DLAT and major histocompatibility complex (MHC) genes in all cancer types, particularly in KIRC, LIHC, PRAD, TGCT, and UVM. In addition, immunosuppressive genes and immune activation genes were both strongly linked to DLAT expression.

## DISCUSSION

Cuproptosis is a recently discovered programmed cell death process that is induced by copper, as revealed by Tsvetkov et al. [12]. Despite this, the role of cuproptosis in the tumorigenesis and progression of cancer is still unclear. DLAT, a key molecule in cuproptosis whose accumulation in mitochondria directly triggers this process, has been identified as a potential candidate for further exploration [12]. Therefore, in this study, we conducted a comprehensive investigation into the expression of DLAT and its prognostic implications, while also exploring its potential relationship with tumor immunity in the context of pan-cancer analysis.

We initiated our study by conducting an in-depth analysis of TCGA datasets to ascertain the expression profiles of DLAT and its prognostic significance. Our results revealed that the mRNA level of DLAT was significantly elevated in seven types of cancer, namely, CHOL, ESCA, LIHC, LUAD, LUSC, STAD, and STES, in comparison to both paracancerous and normal tissues. In contrast, DLAT expression was lower in BRCA, COAD, COADREAD, HNSC, KIPAN, KIRC, KIRP, PRAD, and THCA. Notably, these findings are consistent with earlier reports in patients with CHOL and STAD [13, 14]. Furthermore, we corroborated our findings with IHC analysis from the HPA database. Previous research has revealed that DLAT could promote tumor cell progression in gastric cancer [13]. According to the results of the KM analysis, our research suggested that increased DLAT expression had an association with dismal OS in patients suffering from BRCA, GBMLGG, LGG, LIHC, and PAAD, while opposite results were found in COADREAD, KIPAN, KIRC, KIRP, and READ. These results offer strong support that DLAT could function as a possible biological marker for inferring the prognosis of patients with tumors.

One further significant discovery made by this research is that DLAT is closely related to cancer immunity. In recent years, a growing body of research has demonstrated that the immune status of tumors is closely linked to the cellular composition and infiltration levels in their corresponding microenvironments [15–17]. The ESTIMATE algorithm has been shown to be a convenient and efficient method for predicting tumor purity, which is a reflection of the characteristics of the TME, and has been identified as a prognostic factor in various human malignancies, particularly colon cancer [18]. By analyzing the TCGA cohort, we discovered that DLAT had a strong inverse association with the TME immune composition in 27 different cancers, namely ACC, BRCA, CESC, ESCA, GBM, GBMLGG, HNSC, LAML, LGG, LUAD, LUSC, MESO, KIPAN, KIRC, KIRP, PCPG, PRAD, SARC, SKCM, SKCM-M, SKCM-P, STAD, STES, TGCT, THCA, THYM, and UCEC, and inversely linked to the TME stromal component in 14 cancers, notably, ACC, CESC, ESCA, GBM, KIPAN, LUAD, LUSC, PCPG, SARC, SKCM-P, STAD, STES, THCA, and UCEC. Furthermore, our GSEA analysis revealed a strong association between DLAT and immune-related pathways, particularly the B-cell receptor (BCR) signaling pathway, which is critical for normal B cell maturation and adaptive immunity. Gunderson et al. also reported that the BCR signaling pathway plays a vital role in the pancreatic cancer microenvironment [19]. Moreover, we have uncovered a significant association between DLAT and immune cell infiltration levels in multiple cancer types, as determined by the TIMER algorithm. Notably, results from the CIBERSORT algorithm showed a strong link between DLAT expression and TAMs, which are the most abundant immune cell component in TME and can differentiate into two subtypes: M1 and M2 [20]. M1 macrophages play a vital role in innate defense and eliminating cancerous cells, thus being recognized as macrophages with anti-cancer or “good” properties [21]. Intriguingly, DLAT exhibited a positive correlation with M1 macrophages in COADREAD, KIPAN, KIRC, and READ, partially explaining its protective function for overall survival in these cancer types. In addition, we discovered that genes that encode chemokines, chemokine receptor proteins, MHC, immunostimulators, and immune inhibitors were co-expressed with the DLAT gene. Overall, these findings provide new insights into the potential involvement of DLAT in modulating the immune response in cancer and suggest that DLAT may be a potential target for immunotherapy.

TMB is a hopeful prognostic biological marker that has the potential to assist in immunotherapeutic interventions [22, 23]. Earlier studies have demonstrated that TMB may be employed as a biological marker to improve the effectiveness of immunotherapy for treating multiple cancers [24–26]. MSI is also an important biological marker in ICIs therapy [25, 27]. In colorectal cancer, the presence of high-frequency MSI is a predictor of clinical features and prognosis that is independent of other factors [28]. The results of our research indicated that DLAT expression was linked to TMB in 10 different cancers and MSI in 11 different cancers. This could be an indication that the degree of DLAT expression might alter the MSI and TMB of the tumor, which will influence the patient’s responsiveness to ICI therapy. Such findings will serve as a new standard for determining immunotherapy outcomes.

Despite the integration of information from various databases, our study still has some limitations. Firstly, although the bioinformatic analysis has provided us with meaningful insights regarding DLAT’s role in cancer, biological experiments *in vitro* or *in vivo* are necessary to validate our findings and translate them into clinical practice. Moreover, further mechanistic studies are needed to better understand the molecular and cellular functions of DLAT in cancer. Secondly, relying solely on a single-gene marker is insufficient for predicting patient outcomes or as biometric features. The use of network or sub-network markers is necessary to increase the accuracy and precision of predictions. For instance, Song et al. proposed a method to identify survival prognostic subnetwork signatures that incorporated gene expression patterns, representative protein-protein interactions, and clinical metastatic potential [29]. This approach has been shown to be more accurate and effective in predicting survival time without distant metastases.

In conclusion, our research revealed the expression as well as the prognostic landscape of DLAT in pan-cancer. We also found a strong link between DLAT expression and the infiltration level of immune cells, especially TAMs. Additionally, DLAT expression was demonstrated to be linked to both TMB and MSI in various cancers; this suggests that DLAT is related to existing predictors for the effectiveness of ICIs. The recent advancements in single-cell technologies have led to the proliferation of single-cell multi-omics data. For instance, Song et al. have developed a novel method known as the Single-cell Multi-omics Gene co-Regulatory algorithm, which utilizes scATAC-seq and scRNA-seq data to detect target genes and coherent functional regulatory signals [30]. Therefore, in the future, the precise and reliable integrative analysis of single-cell multi-omics data might aid in uncovering the inherent molecular foundations and underlying mechanisms of DLAT in cancer.

## MATERIALS AND METHODS

### Data processing and differential expression analysis

RNA sequencing and associated phenotype data were extracted from TCGA (a total of 10535 samples) utilizing UCSC Xena (https://xena.ucsc.edu/). In addition, DLAT expression data from tumor cell lines and normal tissues were acquired from the CCLE database (https://portals.broadinstitute.org/ccle/) and the GTEx database (https://commonfund.nih.gov/GTEx) respectively for a multidimensional investigation.

Cancer types with fewer than three normal samples in the TCGA database were excluded for a robust differential expression analysis. Finally, the DLAT expression levels in 26 distinct human cancer types were analyzed. The R package “ggpubr” was employed to visually display the data.

### Immunohistochemistry staining

The IHC data of DLAT expression were extracted and analyzed based on the HPA database (http://www.proteinatlas.org/).

### DLAT gene expression and survival

To explore the potential prognostic value of DLAT in cancer, the survival data of OS, DSS, DFI, and PFI downloaded from TCGA were analyzed. For each different form of cancer, we used both the Cox regression analysis and the KM survival method to analyze the survival data. The threshold value for categorizing patients into two groups was determined according to each tumor’s median value of DLAT expression. The R packages “forestplot,” “survminer,” and “survival” were employed to visually represent the data.

### DLAT genetic alteration analysis

To explore DLAT genomic alterations across the TCGA cancer types, we applied the “Cancer Types Summary” section of the cBioPortal website (www.cbioportal.org) [31]. In addition, the methylation data (merged HM27 and HM450), as well as the CNV data, were also downloaded from the cBioPortal website. The R package “ggpubr” was used to analyze and visually display the relationship between the levels of DLAT expression and its promoter methylation levels or CNV.

### Tumor mutation burden and microsatellite instability

TMB refers to the overall amount of somatic coding mutations present in a particular kind of cancer and has been shown to correlate highly with immunotherapy’s efficacy [32–34]. We estimated each patient’s TMB score after downloading the data on somatic mutations from the UCSC Xena database, which included information on all TCGA patients. MSI, which suggests a deficiency in DNA mismatch repair, is a biomarker that indicates favorable responsiveness to immunotherapy [35]. The MSI data in this research was extracted from a published study [36]. As part of this research, we determined if there was a connection between DLAT expression and TMB or MSI in various cancers. The “ggpubr” R package was utilized for visualizations of the results.

### Association of DLAT expression with immunity

ESTIMATE is an algorithm to infer the abundance of immune and stromal components within tumors. With this study, the R package “estimate” was used to calculate the ImmuneScore and StromalScore of each tumor sample. Furthermore, we used two algorithms, including TIMER and CIBERSORT, to analyze the link between DLAT expression and the infiltration of different immune cell types in various tumors. Additionally, we utilized the “limma” R package to carry out co-expression analyses of DLAT and immune-related genes, including those encoding chemokines, chemokine receptor proteins, MHC, immunostimulators, and immune inhibitors. To display the findings, the “ggpubr,” “reshape2,” and “RColorBreyer” packages were utilized.

### Gene set enrichment analyses

DLAT’s biological functions in tumors were investigated via GSEA. The R package “clusterProfiler” and Reactome gene sets obtained through the GSEA website (https://www.gsea-msigdb.org/gsea/downloads.jsp) were applied for GSEA.

### Statistical analysis

Expression data in this study were Log2(x+1) transformed. T-tests were utilized to estimate the differences in levels of DLAT expression between tumor and normal samples. The differential analyses of survival outcomes between high- and low-expression of DLAT were conducted using the log-rank test. The relationship between two variables was examined using Spearman’s test. All statistical analyses were done in R (Version 4.0.3). The statistical significance level was set at p < 0.05.

## Supplementary Material

Supplementary Figures
