# Supplementary material for: Systematic pan-cancer analysis identifies cuproptosis-related gene DLAT as an immunological and prognostic biomarker
Source: Aging (Albany NY). 2023 May 17;15(10):4269–87. doi: 10.18632/aging.204728 (PMC10258010; doi:10.18632/aging.204728)
Supplement: Supplementary Figures [file aging-15-204728-s001.pdf]

SUPPLEMENTARY FIGURES

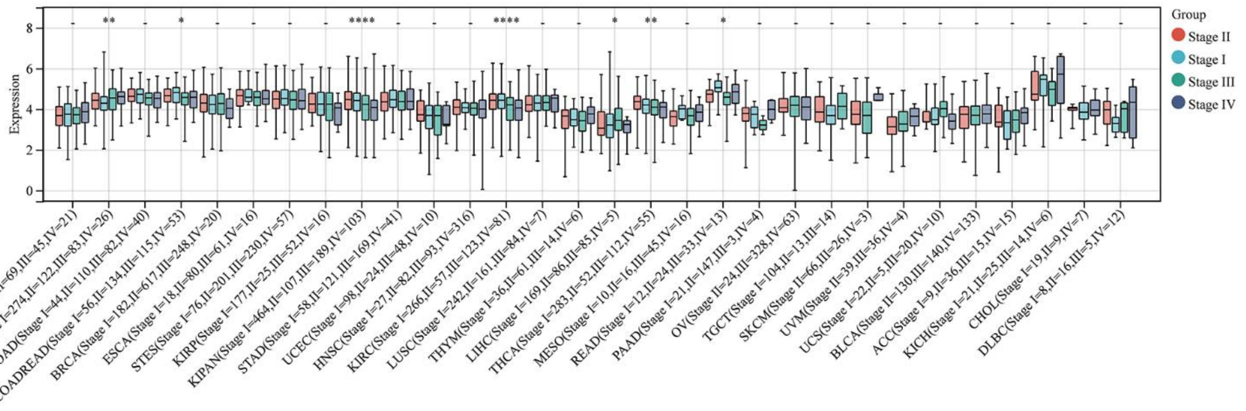

Supplementary Figure 1. Pan-cancer DLAT expression in different stages. -, not significant, *p < 0.05, **p < 0.01, ***p < 0.001 and ****p < 0.0001.

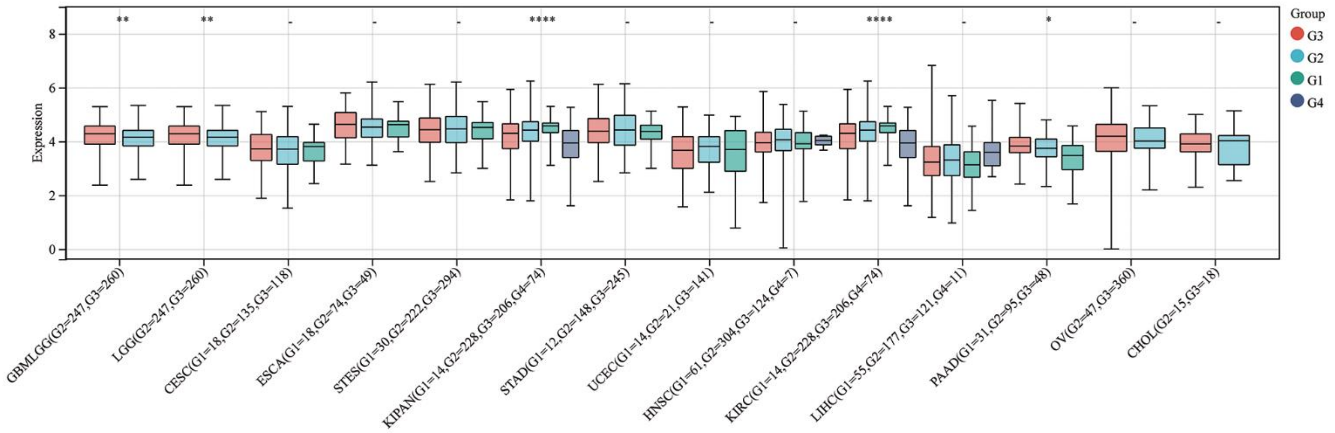

Supplementary Figure 2. Pan-cancer DLAT expression in different grades. -, not significant, *p < 0.05, **p < 0.01, ***p < 0.001 and ****p < 0.0001.
